# Simultaneous clinical resolution of focal segmental glomerulosclerosis associated with chronic lymphocytic leukaemia treated with fludarabine, cyclophosphamide and rituximab

**DOI:** 10.1186/1471-2369-12-33

**Published:** 2011-07-05

**Authors:** Spyridon Arampatzis, Nikolaos Giannakoulas, Vassilios Liakopoulos, Theodoros Eleftheriadis, Panagiota Kourti, Foteini Karasavvidou, Panagiota Matsouka, Ioannis Stefanidis

**Affiliations:** 1 Department of Nephrology, University of Thessaly, Larissa, Greece; 2 Department of Haematology, University of Thessaly, Larissa, Greece; 3 Department of Pathology, University of Thessaly, Larissa, Greece

**Keywords:** chronic lymphocytic leukemia, focal segmental glomerulosclerosis, nephrotic syndrome, fludarabine, cyclophosphamide, rituximab

## Abstract

**Background:**

Although renal involvement in advanced haematological malignancies is common, glomerulonephritis associated with lymphoproliferative disorders is rare, and the related pathogenetic mechanisms are still poorly understood. We present a rare case of chronic lymphocytic leukaemia(CLL)-associated focal segmental glomerulosclerosis with nephrotic-range proteinuria.

**Case presentation:**

A 53-year-old Caucasian man, previously healthy, with no history of hypertension, alcohol use or smoking presented with rapid weight gain, massive peripheral oedema, and hypertension. Laboratory findings included a white blood cell count of 49,800 cells/mm^3 ^with an absolute lymphocyte count of 47,000 cells/mm^3^, serum albumin of 2.3 g/dL, urea 65 mg/dL, and creatinine 1.5 mg/dL. A 24-hour urine collection contained 7.1 g protein and significant haematuria. A peripheral blood smear showed mature lymphocytosis and smudge cells. Diagnostic imaging showed mild paraaortic lymphadenopathy with no renal abnormalities. Bone marrow aspiration and trephine biopsy showed diffuse and focal infiltration with B-CLL lymphocytes. Percutaneous renal biopsy revealed total sclerosis in 3/21(14%) of the glomeruli and focal and segmental solidification and sclerosis in 4/21 (19%) glomeruli. A regimen of fludarabine, cyclophosphamide and rituximab was successful in inducing remission of the CLL and clinical resolution of the nephritic-range proteinuria.

**Conclusions:**

A multidisciplinary approach to monitor both the malignancy and the glomerular lesions is crucial for the optimal management of paraneoplastic glomerulonephritis. Although chemotherapy with fludarabine, cyclophosphamide and rituximab successfully treated CLL-associated nephrotic syndrome in our patient, further studies are required to confirm efficacy in this setting.

## Background

Although renal involvement in advanced haematological malignancies is common, glomerulonephritis associated with lymphoproliferative disorders is rare, and the related pathogenetic mechanisms are still poorly understood [1]. Chronic lymphocytic leukemia (CLL) is more commonly associated with membranoproliferative glomerulonephritis and membranous nephropathy whereas minimal change disease is the most common paraneoplastic glomerulonephritis associated with Hodgkin lymphoma, followed by focal segmental glomerulosclerosis (FSGS) [2]. We report on a patient with CLL who presented with NS-associated FSGS - a rare association.

## Case presentation

**Figure 1:**
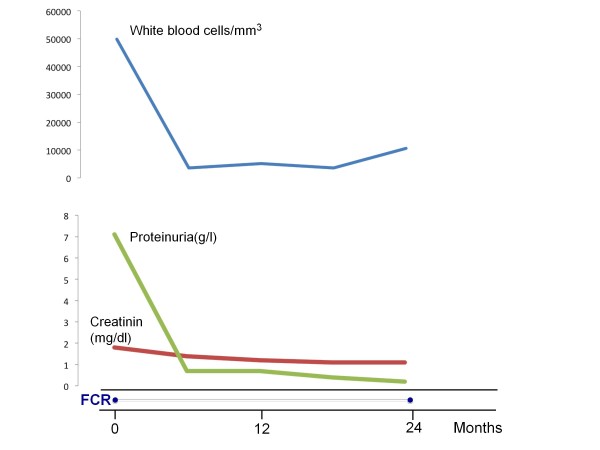
**Laboratory values and white blood cell count before and after treatment with fludarabine, cyclophosphamide, and rituximab (FCR)**.

A 53-year-old Caucasian man, 78 kg, 1.67 cm, previously healthy, with no history of hypertension, alcohol use or smoking, presented with a rapid 5 kg weight gain in the past week, massive peripheral oedema, hypertension (170/90 mmHg) and leukocytosis. He had no peripheral lymphadenopathy or organomegaly. A haemogram showed a white blood cell (WBC) count of 49,800 cells/mm^3 ^with an absolute lymphocyte count (ALC) of 47,000 cells/mm^3 ^(Figure 1), haemoglobin of 11.4 g/dL, and platelet count of 314,000 cells/mm^3^. A peripheral blood smear showed mature lymphocytosis and smudge cells. Flow cytometry of peripheral blood lymphocytes showed a clonal B-cell population (CD20+, CD79b+, CD5+, CD23+, CD43+, CD11c-, FMC7-/+, CD38-, ZAP-70 29%, ck/cλ = 66) consistent with CLL (CLL score 4). Serum albumin was 2.3 g/dL, urea 65 mg/dL, creatinine 1.5 mg/dL, cholesterol 348 mg/dL, and beta2 microglobulin 5.39 mg/L. Hypoglobulinaemia was present with IgG 307 mg/dL (normal range 847-1690 mg/dL), IgA 71 mg/dL (normal range 99-300 mg/dL), IgM 59 mg/dL (normal range 64-249 mg/L. No monoclonal component was found in serum analysis (serum-free chain k 22.9 mg/L [normal range 3.3-19.4 mg/L], λ 16 mg/L [normal range 5.71-26.3 mg/L], k/λ-quotient 1.43 [normal range 0.26-1.65]). Urine electrophoresis failed to identify monoclonal protein excretion (urine light-free chain k was 34.9 mg/L, and λ was 14.2 mg/L). Kidney ultrasound showed no abnormal findings. 24-hour urine cyewqollection contained 7.1 g of protein and significant haematuria.

**Figure 2:**
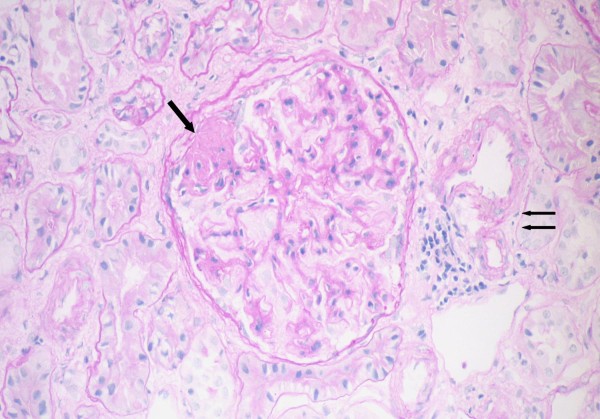
**Directly opposite to the vascular pole (double arrow), a segmental lesion of sclerosis (single arrow) that adheres to Bowman's capsule, which shows significant, wrinkling (Pas stain × 400)**.

**Figure 3:**
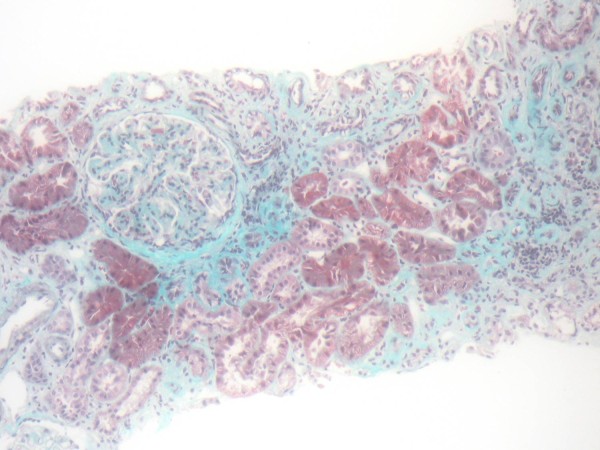
**Chronic interstitial nephritis, interstitial fibrosis, mild chronic inflammation and tubular atrophy**. The glomerulus here is enlarged and shows diffuse, mild to moderate mesangial expansion and hypercellularity without segmental sclerosis. (Masson trichrome stain × 200).

A CT scan of the thorax and abdomen showed mild paraaortic lymphadenopathy. Bone marrow aspiration and trephine biopsy showed a diffuse and focal infiltration with B-CLL lymphocytes. Percutaneous renal biopsy was performed with a 16-gauge needle. Twenty-one glomeruli were found in the biopsy cylinder. Three of the glomeruli showed global sclerosis (14%) and 4 revealed segmental and focal solidification and sclerosis of the glomerular tuft (19%), (Figure 2). The non-sclerotic glomeruli showed mild mesangial expansion, and mild to moderate mesangial hypercellularity. The peripheral glomerular capillary wall appeared normal in thickness. The interstitium was expanded by diffuse fibrosis and mild chronic inflammatory cell infiltrations primarily of benign-appearing lymphocytes (Figure 3). The immunofluorescence findings were negative for IgA, IgG, IgM, C3, C4, C1q, kappa and lambda light chains. These findings were consistent with FSGS.

After initial treatment with enoxaparin s.c., valsartan and a statin, the patient received six courses of intravenous fludarabine (25 mg/m^2 ^d1-3), cyclophosphamide (350 mg/m^2 ^d1) and rituximab (375 mg/m^2 ^d1). Each cycle was given every four weeks under continuous prophylactic therapy with acyclovir and trimethoprim-sulfamethoxazole.

The patient showed a gradual response to therapy (Figure 1). One month after the fourth dose, he had complete resolution of oedema, the WBC count had decreased to 3,500/mm^3 ^(ALC: 0.98/mm^3^), and urinary protein excretion had dropped to 0.7/g/day. Serum creatinine was normal at 1.0 g/dL, serum cholesterol had decreased to 195 mg/dL, and serum albumin had increased to 4.4 g/dL. Immunoglobulin and beta2 microglobulin values were normal. After completion of the 6 cycles of therapy, a CT scan of the thorax and abdomen was normal. Bone marrow aspiration and trephine biopsy showed normal haematopoietic cells without clonal B cells. The patient continued to receive rituximab (375 mg/m^2 ^every 3 months) as maintenance therapy for the next 24 months. At the last follow up 28 months after diagnosis the patient remains in complete remission and the urinary protein excretion is below 200 mg/day under antihypertensive treatment.

Fludarabine, cyclophosphamide and rituximab (FCR) therapy is becoming the frontline treatment for CLL, especially in younger patients. These agents act synergistically and are associated with high complete remission rates and long durations of remission [3]. Focal segmental glomerulosclerosis (FSGS) is a glomerular disease characterized by marked proteinuria, hypertension, and a high incidence of progression to renal failure [4]. The pathogenesis of primary FSGS remains unclear but several observations support the hypothesis that there is a causative factor present in the circulation of some patients with FSGS [5]. The recommendations for initial immunosuppressant treatment with prednisone or cyclosporine are largely based upon small clinical studies. Data on the use of cyclophosphamide and other cytotoxic drugs for steroid-dependent or steroid-resistant FSGS in adults are limited to a few retrospective observational studies.

The alkylating agent cyclophosphamide has been primary used in children with relapsing or steroid resistant FSGS. Rituximab is a chimeric CD20-reactive monoclonal antibody with established efficacy in B-cell lymphomas. Binding of the antibody to the CD20 antigen results in selective lysis of targeted B cells. Recently, several case reports demonstrated the successful use of rituximab in a variety of systemic diseases with renal involvement and also in steroid resistant FSGS [6,7]. In several recurrent post-transplant FSGS cases, NS resolved after rituximab treatment [8,9]. Fludarabine, a purine analogue with selective activity against both dividing and resting lymphocytes, results in a profound decrease in CD4 lymphocytes that may last for years. Low-dose fludarabine treatment has been used only in patients with membranous nephropathy [10].

Since FSGS is not an antibody-mediated disease, improvement of NS with the FCR regime may seem surprising. However, B cells play an important role as immunoregulatory cells through antigen processing and presentation, recruitment of auto-reactive T cells, interaction with antigen presenting cells, and secretion of cytokines. B cell depletion under FCR may affect T cell activation and downregulate the costimulation status [11].

The primary site of glomerular injury in FSGS is the podocyte. A variety of factors have been postulated to cause injury to glomerular cells, including circulating cytokines such as permeability altering factor, angiotensin II, macrophage infiltration and other mediators of inflammation [12,13]. Injury to the podocytes by a circulating toxin is further supported by the rapidity and relatively high incidence of disease recurrence in some patients after renal transplantation. Simultaneous presentation and long term remission of CLL and FSGS in our case is consistent with the interpretation that chemotherapy prevented the synthesis of a nephrotoxic circulating lymphokine secreted by malignant cells. A potential paraneoplastic link between malignancies and glomerulonephropathies is difficult to demonstrate, but is supported in our patient by the parallel improvement and longterm resolution of the two conditions under treatment.

## Conclusion

A multidisciplinary approach to monitor both the malignancy and the glomerular lesions is crucial for the optimal management of paraneoplastic glomerulonephritis [2]. Although chemotherapy with FRC successfully treated CLL-associated nephrotic syndrome in our patient, further studies are required to confirm efficacy in this setting.

## Competing interests

The authors declare that they have no competing interests.

## Authors' contributions

SA, NG, VL, ET, PK, PM and IS were the treating physicians. FK evaluated the renal biopsy. All of the authors contributed to the preparation of the manuscript and have read and agree to the manuscript as written.

## Pre-publication history

The pre-publication history for this paper can be accessed here:

http://www.biomedcentral.com/1471-2369/12/33/prepub
